# MIP3α as an early prognostic predictor for patients with B-cell malignancies receiving CD19/CD22-redirected CAR-T cell cocktail therapy

**DOI:** 10.1007/s00262-023-03418-2

**Published:** 2023-03-04

**Authors:** Jin Jin, Tianjiao Liu, Jiali Cheng, Jiao Meng, Na Wang, Liang Huang, Xiaoxi Zhou, Liting Chen, Hui Luo, Jianfeng Zhou

**Affiliations:** 1 Department of Hematology, Tongji Hospital, Tongji Medical College, Huazhong University of Science and Technology, Wuhan, Hubei China; 2 Department of Hematology, Cancer Hospital of Harbin Medical University, Harbin, Heilongjiang China

**Keywords:** Chimeric antigen receptor-T therapy, Relapse, Cytokines, Biomarkers, MIP3α, T-cell infiltration

## Abstract

**Purpose:**

Identifying the temporal pattern of recurrence and prognostic biomarkers would further help improve the efficacy of chimeric antigen receptor (CAR) -T therapy.

**Methods:**

We examined the prognoses of 119 patients after sequential infusion of anti-CD19 and anti-CD22, a cocktail of 2 single-target CAR (CAR19/22) T cells in an open-label, single-center clinical trial (ChiCTR-OPN-16008526). And we, from a 70-biomarker panel, identified candidate cytokines that might predict the treatment failure, including primary non-response (NR) and early relapse (ER).

**Results:**

In our study, 3 (11.5%) patients with B-cell acute lymphoblastic leukemia (B-ALL) and 9 (12.2%) cases of B-cell non-Hodgkin lymphoma (NHL) failed to respond to sequential CAR19/22 T-cell infusion (NR). A total of 11 (42.3%) B-ALL patients and 30 (52.7%) B-NHL patients had relapses during follow-up. Most recurrence events (67.5%) occurred within six months of sequential CAR T-cell infusion (ER). We found that macrophage inflammatory protein (MIP)-3α was a highly sensitive and specific prognostic predictor for patients with NR/ER and those attaining over-6-month remission. Patients who had higher MIP3α levels after sequential CAR19/22 T-cell infusion had significantly favorable progression-free survival (PFS) than their counterparts with relatively lower MIP3α expression. Our experiments demonstrated that MIP3α could enhance the therapeutic effect of CAR-T cells by promoting T-cell infiltration into and enriching memory-phenotype T cells in the tumor environment.

**Conclusion:**

This study showed that relapse occurred mainly within six months after sequential CAR19/22 T-cell infusion. Moreover, MIP3α could act as a valuable post-infusion biomarker for identifying patients with NR/ER.

**Supplementary Information:**

The online version contains supplementary material available at 10.1007/s00262-023-03418-2.

## Introduction

Immunotherapy, especially CAR-T therapy, has ushered in a new era of tumor treatment [1–5]. With this novel treatment modality, the complete response (CR) rate of hematological malignancies can reach up to 90% [1, 2]. Nonetheless, mounting evidence indicated that patients receiving CAR-T cells might only attain short-term remission. A long-term follow-up of 19-28z CAR-T products for the treatment of B-ALL demonstrated that even if the CR rate was 83%, 65.4% (17/26) of patients, without further intervention, developed a relapse [3]. And this ratio arrived at 48% (12/25) in the phase II clinical trial of ZUMA-3 [6]. Antigen-negative escape represents one of the most common causes of recurrence in single-target CAR-T therapy, and most studies showed that dual-target CAR T-cell strategies could prevent antigen downregulation, which might reduce relapse by decreasing the antigen-negative escape [7, 8]. Our center conducted a clinical trial of sequential infusion of a cocktail of anti-CD19 and anti-CD22 CAR (CAR19/22) T cells. While 96% of B-ALL patients and 50% of B-NHL patients accomplished CR, virtually half of the patients later experienced CD19/CD22-double-positive relapse [2]. More active subsequent therapies such as hematopoietic stem cell transplantation (HSCT) [1, 9, 10], checkpoint blockade therapy [11], etc. can be employed to improve the prognosis of patients who are prone to early relapse after CAR T-cell therapy. Therefore, identifying patients at higher risk of non-response or relapse by means of clinical features and biomarkers at an earlier time point is clinically relevant, since such screening can allow for sufficient time to bridge other treatments. Such identification can lead to early intervention for high-risk patients and save low-risk patients from over-treatment and related adverse reactions. Knowing when relapse will take place and who is susceptible to relapses can further improve the efficacy of CAR19/22 T-cell cocktail therapy.

Currently, multiple studies are endeavoring to identify factors that are related to the clinical response to CAR-T therapy. Carl H. June’s team found that less differentiated T-cell subsets (CD45RO-CD27 + CD8 +) in CAR-T products before infusion might be a predictor of prognosis, and the proportion of this memory-phenotype cells was significantly higher in CR patients than in their partially responding (PR)/NR counterparts [12]. Another clinical trial on CD19-redirected CAR-T cells (NCT01865617) demonstrated that serum LDH, MCP-1 and interleukin (IL)‐7 were independently related to PFS in B-NHL patients [13]. CAR transgene copy numbers [14, 15] and B cell aplasia [16] could also partially reflect the therapeutic effect of CAR-T cells. Up till now, no biomarkers indicative of the effect of CAR19/22 T-cell cocktail therapy are available.

In this study, we examined the features of recurrent patients with B-cell malignancies after sequential infusion of CAR19/22 T cells and found that the majority (67.5%) of recurrent events occurred within six months of sequential CAR T-cell infusion, and patients on remission for more than 6 months relapsed less. Moreover, we found that MIP3α could serve as a prognostic predictor with high sensitivity and specificity for identifying patients at higher risk for non-response/early relapse (NR/ER) at an earlier time point, thereby providing guidance for early intervention after CAR-T therapy.

## Materials and methods

### Patients

Between October 2017 and August 2019, we recruited 119 consecutive patients with aggressive B-cell malignancies who had been involved in the clinical trial of sequential infusion of anti-CD19 and anti-CD22 CAR-T therapy (ChiCTR-OPN-16008526 at http://www.chictr.org.cn) [2]. All patients provided informed consent in strict accordance with the Declaration of Helsinki.

### Detection of cytokine production

Since the onset of CRS varied among different patients, we collected serial serum samples during the first 30 days after sequential CAR T-cell infusion and retrospectively obtained a total of 73 serum specimens at the peak of serum IL-6 level [17]. The 35 baseline serum samples of patients and 3 serum specimens from healthy donors were also harvested. The serum specimens during the training phases were evaluated by a 70-biomarker panel (Meso Scale Discovery, Germany, Cat. K1508K) as previously described [17]. The MIP3α level in serum samples of the validation cohort was determined by employing Bio-Plex Pro Reagent Kit (Bio-Rad Life Science, Hercules, CA, USA) on a Luminex FlEXMAP 3D system (Luminex, Austin, TX, USA) according to the manufacturer’s protocols. Relative changes (*versus* healthy donors) were calculated and the Mann–Whitney test was used for the inter-group comparison.

### Statistical analysis

SPSS 25.0 software package was used for data analysis. Unless otherwise stated, all the data were representative of the results of at least three independent experiments.

## Results

### Patient characteristics

Between October 2017 and August 2019, a total of 119 consecutive patients who had been on the CAR19/22 T-cell cocktail therapy clinical trial (ChiCTR-OPN-16008526) were retrospectively enrolled in this study, including 35 B-ALL patients and 84 B-NHL patients. The baseline characteristics of these patients were summarized in Table 1 and supplementary Table 2. Fifty patients (41.0%) were female, and the median age was 44 years (range, 11–67 years) with 93.5% aged ≥ 18 years. All patients were refractory (29.4%) or relapsed (70.6%) after their prior therapy; 58 patients (48.7%) had been subjected to ≥ 3 lines of treatment. Tumor cells were found in the central nervous system of 13 patients (10.9%). 21.8% of patients had previously received autologous (n = 18) or allogeneic (n = 8) HSCT.

**Table1:** Clinical characteristics of 119 patients in our cohort

	B-ALL (n = 35)	B-NHL (n = 84)
Age		
Years, median (range)	33 (11–60)	47 (17–67)
Sex		
Female	22 (62.9)	28 (33.3)
Prior HSCT		
Autologous	2 (5.7)	16 (19.0)
Allogeneic	7 (20.0)	1 (1.2)
Prior treatment		
First line	9 (25.7)	10 (11.9)
Sencond line	15 (42.9)	27 (32.1)
≥ Third line	11 (31.4)	47 (56.0)
Refractory or relapsed		
Refractory	10 (28.6)	25 (29.8)
First relapse	15 (42.9)	23 (27.4)
≥ Second relapse	10 (28.6)	36 (42.9)
CNS involvement		
Yes	4 (11.4)	9 (10.7)
Genetic mutation		
TP53 mutation	5 (14.3)	31 (36.9)
CAR-T cell dose, × 10^^6^ cells/kg, median (range)		
CAR19 T cells	3.04 (1.00–10.00)	4.55 (1.35–10.00)
CAR22 T cells	3.41 (1.00–10.00)	5.77 (1.90–11.35)
Inflammatory factor level		
IL-6 max, pg/ml, median (range)	269.8 (15.73–5000)	214.8 (9.71–5000)
Time to IL-6 peak, days, median (range)	5 (2–9)	5 (2–10)
Ferritin max, μg/L, median (range)	1083.5 (77.4–50,000)	845.1 (174.7–50,000)
CRS grade		
Grade 0–2	30 (85.7)	76 (90.5)
Grade 3–5	5 (14.3)	8 (9.5)
Outcomes		
No response	3 (8.6)	9 (10.7)
Relapse	11 (31.4)	30 (35.7)
Remission during follow-up	12 (34.3)	35 (41.7)
Excluded from response evaluation	9 (25.7)	10 (11.9)
Bridge to HSCT	7 (20)	4 (4.8)
Loss to follow-up	2 (5.7)	2 (2.4)
Non-recurrent deaths^*^	0 (0)	4 (4.8)
Follow-up time		
Days, median (range)	265 (9–988)	192 (25–1005)

^*^The death was caused by thrombocytopenia complicated by hemorrhage, persistent hepatic dysfunction, infection, and intestinal obstruction, respectively

All patients were given CAR19/22 T-cell cocktail infusion after lymphodepletion. The median total dose of CAR19 T cells was 4.1 ± 2.1 × 10^^6^/kg, and that of CAR22 T cells was 5.1 ± 2.5 × 10^^6^/kg. A total of 117 patients (98.3%) developed CRS; only 13 patients (10.9%) were rated ≥ grade 3. The cutoff date for data collection was 30 August 2020, and the median follow-up time lasted 208 days (range, 9–1005). The 12-month OS in B-ALL and B-NHL was 78.86% (95% CI 60.70–89.32%) and 53.06% (95% CI 41.81–63.10%), respectively, and the 12-month PFS in B-ALL and B-NHL was 69.89% (95% CI 51.24–82.53%) and 49.30% (95% CI 38.15–59.51%), respectively (supplementary Fig. 3a-b). Nine B-ALL patients and 10 B-NHL patients were not subject to the response evaluation due to the development of HSCT (n = 11), non-recurrent deaths (n = 4) or loss to follow-up (n = 4) within 6 months after CAR T-cell infusion.

**Fig. 1:**
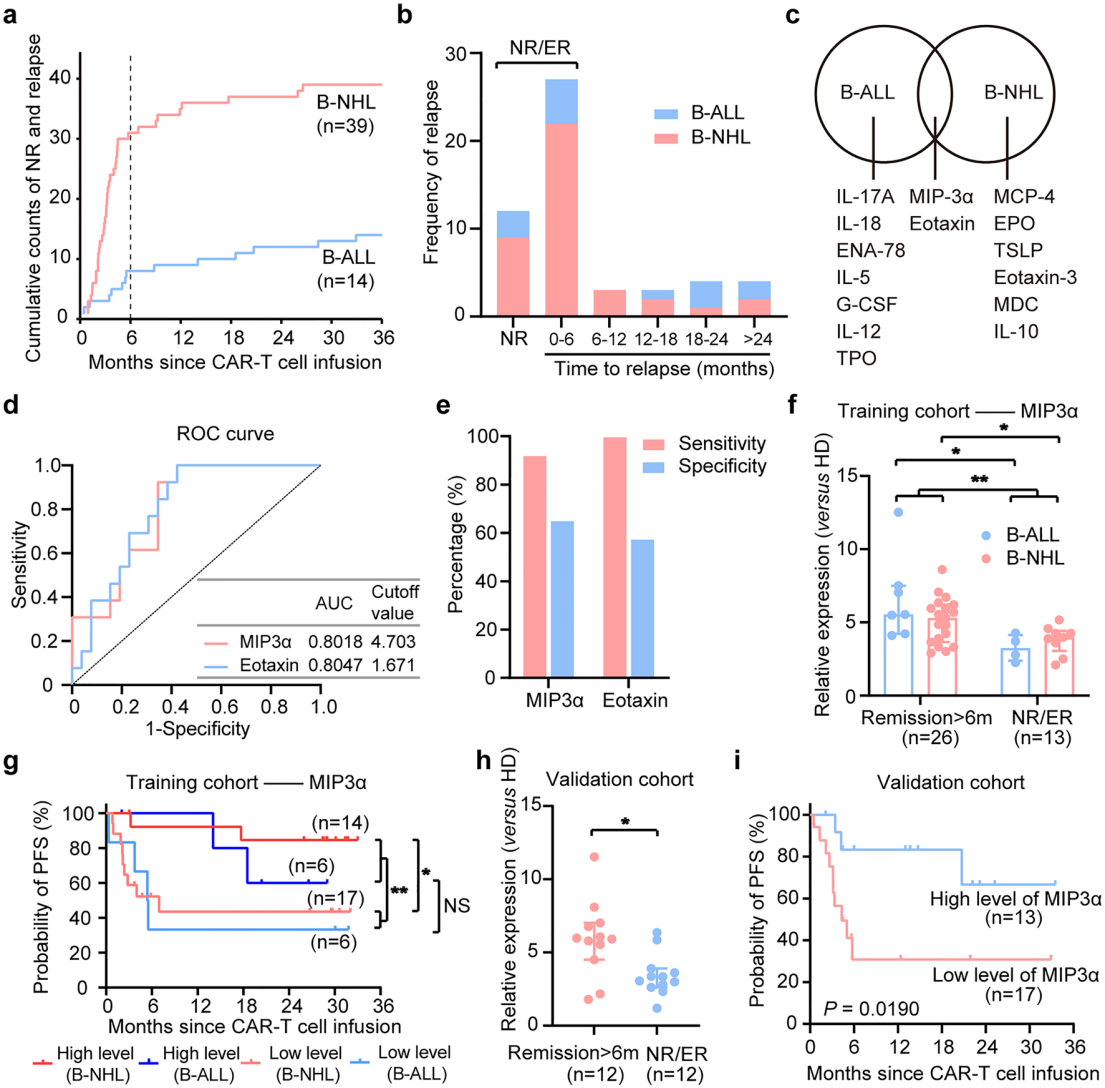
Screening of cytokines associated with prognosis, **a** the cumulative event curve of non-response and relapse events among B-ALL patients (n = 35) and B-NHL patients (n = 84). **b** Frequency of non-response and relapse events per 6 months after CAR19/22 T-cell infusion. **c** Biomarker screening was performed in B-ALL and B-NHL patients, respectively, and cytokines with significant differences are shown in the Venn diagram. **d** To assess the predictive power of MIP3α and Eotaxin, ROC analysis was performed in the training group. **e** The sensitivity and specificity of MIP3α and Eotaxin by using Youden's index. **f** MIP3α showed statistical differences in serum levels between patients with NR/ER and those with over-6-month remission in the training group. **g** The curve of PFS according to MIP3α level in the training group. **h** There were significant differences in serum levels of MIP3α between patients with NR/ER and those with over-6-month remission in the validation group (*P* = 0.0173).  **i** The curve of PFS according to MIP3α level in the validation group

Overall, 11.5% (3/26) of patients with B-ALL and 12.2% (9/74) of patients with B-NHL failed to respond to CAR19/22 T-cell therapy. A total of 11 (42.3%) B-ALL patients and 30 (52.7%) B-NHL patients relapsed during follow-up (Fig. 1a). We further analyzed the time pattern of the relapse after sequential CAR T-cell infusion. In the B-ALL cohort, 5 (45.5%), 0 (0%), 1 (9.1%), 3 (27.3%) and 2 (18.2%) developed a relapse at 6 months, 6–12 months, 12–18 months, 18–24 months and more than 2 years after CAR T-cell infusion, respectively. And the rates in B-NHL patients were 73.3% (22), 10% (3), 6.7% (2), 3.3% (1) and 6.7% (2), respectively. Our results showed that most (67.5%) recurrence events occurred within six months after sequential CAR T-cell infusion, suggesting that post-CAR-T relapse mainly took place at the early stage, and patients in remission for over 6 months were less likely to relapse (Fig. 1b).

### Screening for cytokines associated with prognosis

Clinically, early prediction of non-responsive and relapsed patients can provide guidance for CAR-T therapy. In terms of the temporal pattern of recurrence in our cohort, patients were divided into two groups to identify prognostic biomarkers: the non-response/early relapse (NR/ER) group and the over-6-month remission group. The total proportions of patients with NR/ER in the B-ALL and B-NHL groups were 30.8% and 41.9%, respectively, and 69.2% of B-ALL patients and 58.1% of B-NHL patients achieved remission for more than 6 months. Previous studies indicated that the cytokine levels during the CRS period could partially mirror the function of CAR-T cells in vivo [13, 18]. IL-6 is the cytokine that is most closely correlated with the CRS during the first 30 days after infusion [19]. A total of 73 serum specimens at the peak of IL-6 were retrospectively collected from 119 patients, and 43 of the samples were used as a training cohort to screen prognosis-related cytokines, including 12 B-ALL specimens and 31 B-NHL specimens. These serum samples were examined by using a 70-biomarker panel and the levels of all cytokines (fold change relative to healthy donors) were presented by a heatmap (supplementary Fig. 3c and supplementary Table 2). All cytokines were calculated in terms of the fold changes relative to healthy donors and then analyzed. In the preliminary screening phase, we compared the levels of total biomarkers between patients with NR/ER (n = 13) and patients with over-6-month remission (n = 26) by using Mann–Whitney unpaired test. We screened out 10 cytokines with significant differences between the two groups, i.e., MIP3α, Eotaxin, EPO, TSLP, MCP-4, IL-17F, IL-17A, MDC, TPO, SDF-1a (*P* = 0.002, 0.002, 0.004, 0.008, 0.01, 0.014, 0.025, 0.027, 0.029, 0.032, respectively; supplementary Table 4). Among them, MIP3α was the cytokine with the most significant differences (supplementary Fig. 3d). We then performed cytokine screening in B-ALL and B-NHL patients by using Mann–Whitney unpaired test, respectively, and found that MIP3α and Eotaxin showed statistically significant differences in both B-ALL and B-NHL cohorts (Fig. 1c). Moreover, we found that these two cytokines had a significant linear correlation (r = 0.430, *P* = 0.004, supplementary Fig. 3e). The median fold change of MIP3α (4.273, range, 3.901–5.538) was higher than that of Eotaxin (1.390, range, 0.936–1.912). Considering the *P*-values and fold changes of cytokines (vs. healthy donors), MIP3α was more suitable as a prognostic biomarker than Eotaxin.

To further evaluate the predictive effects of MIP3α and Eotaxin aforementioned, we conducted a receiver operating characteristic (ROC) curve analysis. Both of them were found to have a good predictive power to distinguish patients with NR/ER. The area under each ROC curve (AUC) was 0.8018 (95% CI 0.6657–0.9379), 0.8047 (95% CI 0.6709–0.9386), respectively (Fig. 1d). The cut-off values for fold changes (*versus* healthy donors) of MIP3α and Eotaxin were 4.703 and 1.671 at the peak of serum IL-6 level, respectively, as calculated from *Youden*'s index. The sensitivity of MIP3α and Eotaxin was 92.31% (95% CI 66.69–99.61%) and 100% (95% CI 77.19–100%), respectively. The specificity of MIP3α and Eotaxin was 65.38% (95% CI 46.22–80.59%) and 57.69% (95% CI 38.95–74.46%), respectively (Fig. 1e).

In the training cohort, patients with NR/ER had lower serum MIP3α levels than those with over-6-month remission (*P* = 0.0018, Fig. 1f). Kaplan–Meier analysis showed that patients who had higher MIP3α concentration after sequential CAR T-cell infusion (> 4.703-fold change *versus* healthy donors) had more favorable survival than those with relatively lower MIP3α expression (*P* = 0.0049, Fig. 1g). The same was true of Eotaxin (supplementary Fig. 3f, g). In summary, both MIP3α and Eotaxin were prognosis-related cytokines and could serve as biomarkers for distinguishing between NR/ER and over-6-month remission patients. Considering the balance between sensitivity and specificity, and the fold change compared to healthy donors, we chose MIP3α as a prognostic biomarker. We also determined the baseline MIP3α expression, and found no significant difference between patients with NR/ER and those with over-6-month remission (supplementary Fig. 3 h). Moreover, the expression of MIP3α bore no association with CRS grade and the level of inflammatory cytokines, including IL-6 and ferritin (supplementary Fig. 3i–k), suggesting that the expression of MIP3α might not be affected by the severity of CRS. And the efficacy of CAR-T therapy was not associated with CRS in our cohort (supplementary Fig. 3l).

In order to confirm the predictive power of MIP3α, 10 B-ALL patients and 20 B-NHL patients receiving CD19/CD22-redirected CAR-T cell cocktail therapy were assigned to an independent group for validation. Patients with over-6-month remission also had significantly higher serum MIP3α levels than those with NR/ER in the validation group (*P* = 0.0173, Fig. 1h). Against the cut-off value in the training group, MIP3α had a sensitivity of 83.33% and a specificity of 78.26% in identifying patients with NR/ER (supplementary Fig. 1 m). In addition, extended PFS was also observed in patients with higher serum MIP3α levels in the validation group (*P* = 0.0190, Fig. 1i). Moreover, the baseline characteristics of the training and validation groups were not significantly different (supplementary Table 3). The validation group further proved that MIP3α could sensitively and specifically identify patients with NR/ER on CAR19/22 T-cell immunotherapy.

### MIP3α could recruit more T lymphocytes and enrich the memory phenotype

A previous study on anti-CD19 CAR-T cells (JWCAR029) showed that more infiltrating T cells were enriched in the tumor microenvironment of CR patients compared to their PR counterparts [20]. We thus examined the relationship between the number of infiltrating T cells in tumor tissues and the survival of patients on CAR19/22 T-cell therapy. A total of 10 tumor tissue specimens were obtained from 30 recurrent B-NHL patients. The amount of CD3 + T cells in tumor tissues was found to be intimately related to the PFS, and patients with higher lymphocyte infiltration had protracted survival (*P* = 0.005, r = 0.802, supplementary Fig. 4a, b). In addition, detection of the expression of MIP3α in the peripheral blood of those relapsed patients revealed that patients with less T-cell infiltration might have lower serum MIP3α levels (supplementary Fig. 4c). These results indicated that insufficient T-cell migration was associated with the recurrence after sequential CAR19/22 T-cell infusion, and the expression of MIP3α might be closely related to T-cell infiltration into tumor environment.

**Fig. 2:**
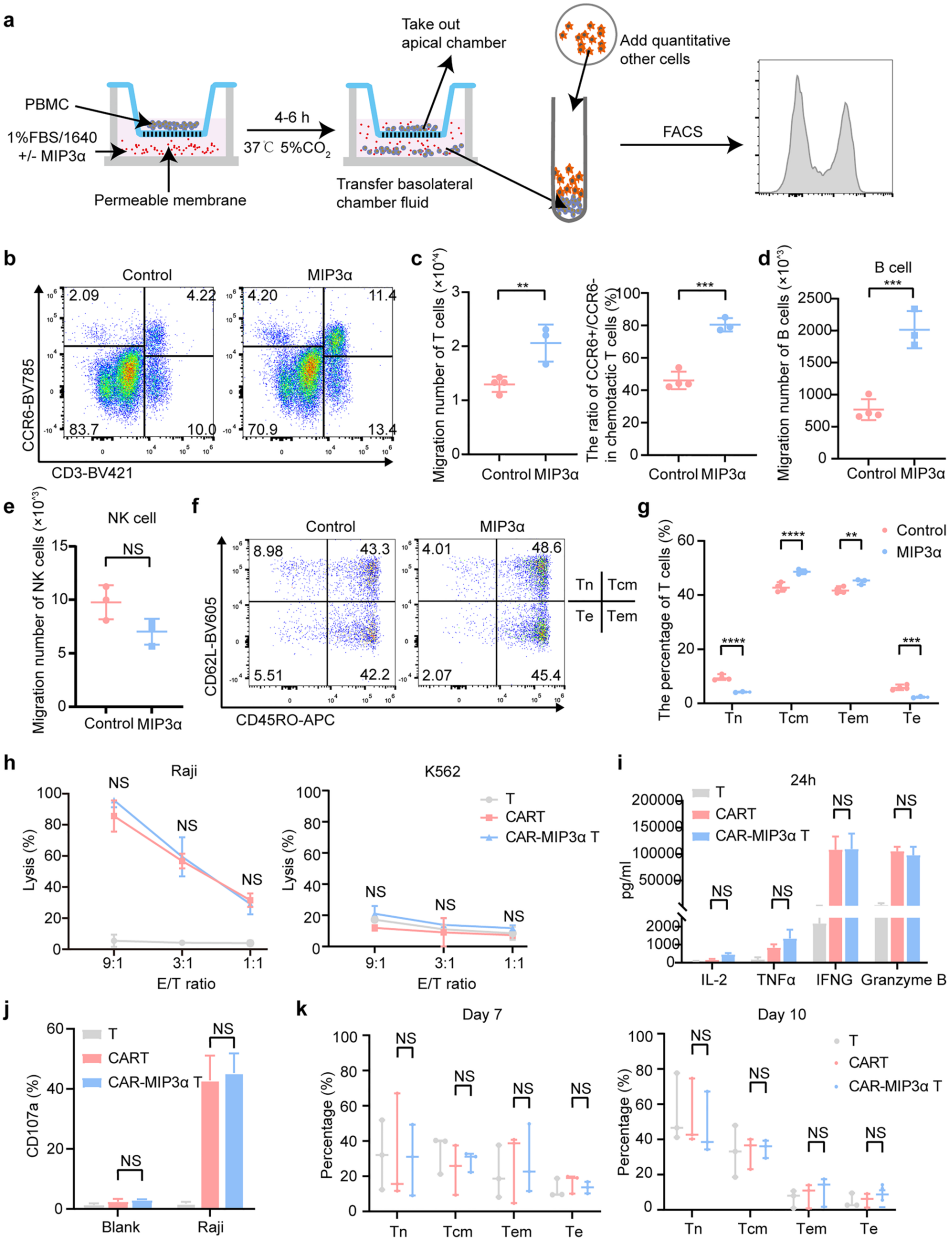
MIP3α could recruit more T lymphocytes and enrich memory phenotype without affecting the anti-tumor effect of CAR-T cells. **a** The graphical representation of the migration assay. **b**, **c** PBMCs in the lower chamber were counted by flow cytometry after incubation for 4 h with MIP3α (1 μg/ml) or culture medium alone. B cells **d** and NK cells **e** of PBMCs in the lower chamber were flow cytometrically counted after incubation for 4 h with MIP3α (1 μg/ml) or culture medium alone. Data represent the average from three donors. **f**, **g** Frequency of central memory (CM; CD62L + CD45RO +), effector memory (EM; CD62L − CD45RO +), effector (CD62L − CD45RO +) and naïve (CD62L + CD45RO −) T cells in recruited T cells. **h** The cytotoxicity of CAR-T/T cells was determined by calcein release assay after 4 h incubated with Raji and K562 cells at different E: T ratios. **i** Quantification of cytokines (IL-2, TNFα, IFN‐γ and Granzyme B) from the supernatant after CAR-T/T cells co-cultured with Raji at an E: T ratio of 1:1 for 24 h. **j** The degranulating effect of CAR-T/T cells co-cultured with Raji cells at an E: T ratio of 1:1 for 4 h. **k** Frequency of CM, EM, effector and naïve T cells in CAR-T/T cells as assessed by using flow cytometry on day 7 and 10. All data represent the average from three donors. Two-way ANOVA was conducted for statistical analysis. NS (not significant): *P* > 0.05, ** *P* < 0.01, *** *P* < 0.001 and **** *P* < 0.0001

MIP3α is a chemokine with a sole specific receptor, CCR6, which is mainly expressed in lymphocytes, especially memory-phenotype T cells (supplementary Fig. 4d, e). In view of this, we conducted in vitro migration experiments to examine the chemotactic effect of MIP3α on PBMCs (Fig. 2a). The results indicated that medium containing MIP3α could significantly attract more T cells (*P* = 0.0092) and B cells (*P* = 0.0008) with high CCR6 expression (Fig. 2b–d), rather than NK cells with low CCR6 expression (Fig. 2e). Meanwhile, the CCR6 level of the T lymphocytes recruited by MIP3α was further increased (*P* = 0.0003, Fig. 2c). Clinical trials on CD19-targeted T cell (CTL019) therapy had indicated that sustained remission was associated with the number of lymphocytes with memory phenotype in CLL patients [12]. Therefore, we also performed the memory-phenotypic analysis of recruited T cells in terms of CD45RO and CD62L expression: central memory (CM; CD62L + CD45RO +), effector memory (EM; CD62L − CD45RO +), effector (CD62L − CD45RO −) and naïve (CD62L + CD45RO −) T cells. Our results showed that the medium containing MIP3α recruited more EM (41.80% vs. 44.97%, *P* = 0.002) and CM (42.85% vs. 48.60%, *P* < 0.0001) T cells compared with the control medium (Fig. 4f-g).

### MIP3α exerted no influence on anti-tumor effects of CAR-T cells

Moreover, it remained unknown whether MIP3α affected the function of T cells. We constructed CAR-MIP3α T cells to express MIP3α (supplementary Fig. 5a, b). The ELISA results showed that MIP3α secreted by CAR-MIP3α T cells was highest in both resting and target cell-based stimulation states (supplementary Fig. 5c). Then, we compared the cytotoxicity and degranulation of conventional CAR- and CAR-MIP3α T cells in vitro, and the results showed that the functions of these two CAR-T cells did not significantly differ (*P* > 0.05, Fig. 2h, j). The cytokines with tumoricidal effect secreted by CAR-T cells, including IL-2, TNFα, IFN‐γ and Granzyme B, were detected by ELISA, and the results revealed no significant differences in these cytokines between CAR- and CAR-MIP3α T cells (*P* > 0.05, Fig. 2i, supplementary Fig. 5d). Moreover, we found that the changes in the phenotypes of T, CAR- and CAR-MIP3α T cells cultured on day 7, 10, 13 and 16 were comparable. The memory subsets in vitro were not related to the MIP3α levels (Fig. 2k, supplementary Fig. 5e). Additionally, the CAR-T cells cultured with different concentrations of MIP3α exhibited no significant differences in functions (supplementary Fig. 5f–h). These findings suggested that MIP3α did not affect the functions of CAR-T cells, in terms of tumoricidal activity, degranulation level, cytokine secretion and population of T-cell subsets.

### MIP3α improved the therapeutic effect of CAR-T cells by increasing T-cell infiltration in vivo

**Fig. 3:**
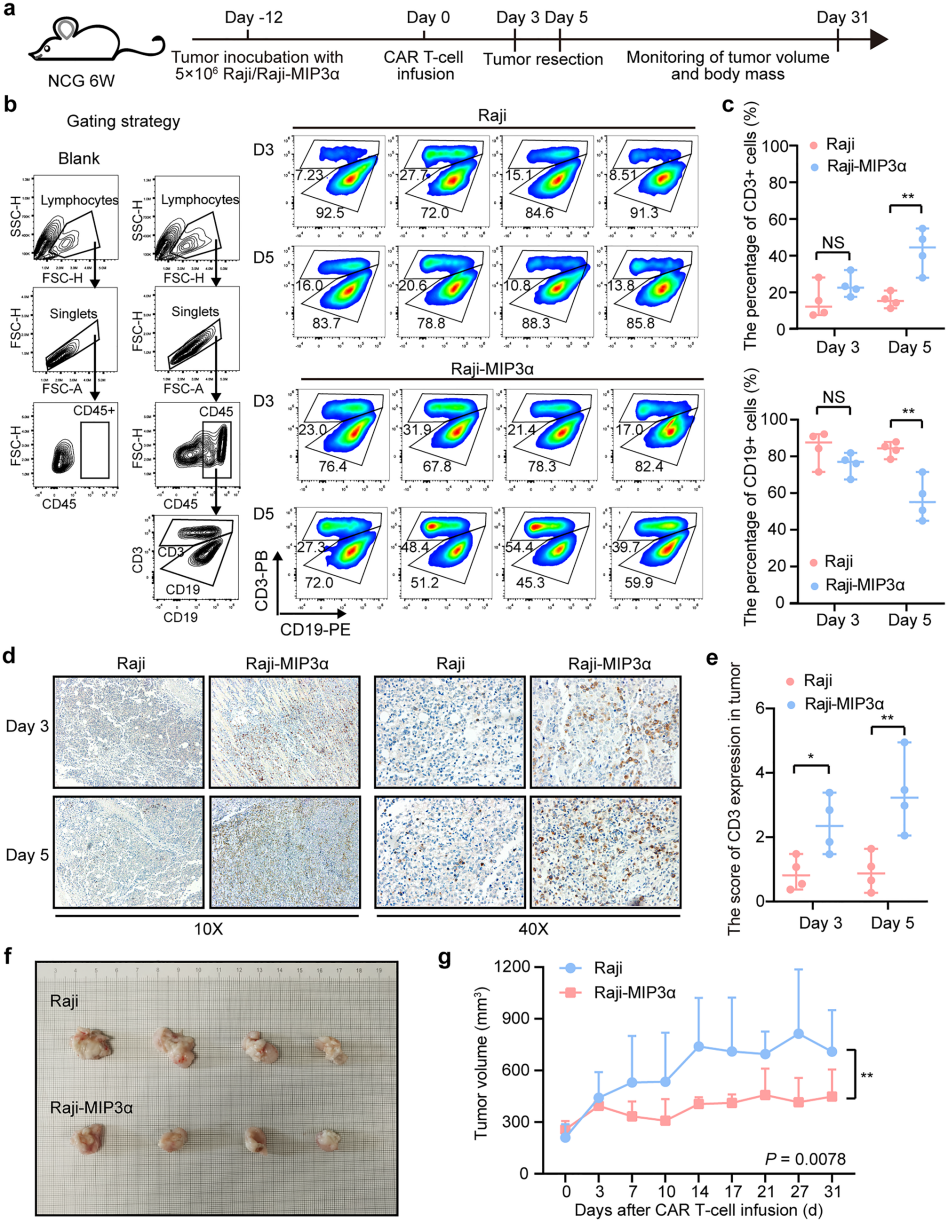
MIP3α improved the therapeutic effect of CAR-T cells in vivo **a** NCG mice were engrafted randomly with Raji or Raji-MIP3α tumor cells. Twelve days after engrafting, the mice were infused with CAR19 T cells via the tail vein. Tumors were resected and tumor-infiltrating T cells were detected on day 3 and day 5. Meanwhile, tumor burden and body mass were regularly monitored in each group, with mice number = 4. **b**, **c** Tumor-infiltrating T cells were flow cytometrically detected. Data represent the mean ± SEM of four mice. **d**, **e** Tumor-infiltrated T cells were detected by IHC. The average density of CD3 positive region was conducted with Image-Pro Plus. Data represent the mean ± SEM of four mice. **f** On 31 days after CAR T-cell infusion, mice from each group were euthanized and tumors were resected, and tumor size was measured. **g** The tumor volume of mice was calculated and the statistical value was analyzed. Two-way ANOVA was conducted for statistical analysis, NS: *P* > 0.05 and ** *P* < 0.01

CAR-T products (CTL019) infused into CR patients after stimulation by cognate CD19 antigen were found to have higher levels of MIP3α than those in PR/NR patients, indicating that CAR-T cells in well-responding patients might release more MIP3α [12]. In order to understand the effect of MIP3α overexpression on CAR-T cells in vivo, we constructed a Raji-MIP3α cell line overexpressing MIP3α. The expression of MIP3α in Raji and Raji-MIP3α cells was found to be 2.309 (2.102–2.516) and 63.119 (59.799–66.439) pg/μl at 24 h, respectively (supplementary Fig. 6a, b), and our results demonstrated that MIP3α had no effect on the proliferation of tumor cells (supplementary Fig. 6c). The lymphoma xenograft tumor model was established by subcutaneous injection of Raji or Raji-MIP3α cells into NCG mice. Tumors were resected at day 3 and 5 after CAR T-cell infusion to analyze infiltrating T lymphocytes (Fig. 3a). Flow cytometry showed that CD3 + T cells conspicuously accumulated in Raji-MIP3α tumors at day 5 compared with Raji tumors, and the median percentage of CD3 positive cells in Raji and Raji-MIP3α tumor sites was 14.64% and 23.33% on day 3, respectively (*P* = 0.3098), and 15.30% and 42.45% on day 5, respectively (*P* = 0.0013). Correspondingly, the median percentage of CD19 positive cells was 85.10% and 76.23% on day 3, respectively (*P* = 0.2900), and 84.14% and 57.10% on day 5, respectively (*P* = 0.0012). From day 3 to 5, a significant increase in tumor-infiltrating T cells was observed in the Raji-MIP3α group (*P* = 0.0200) compared with the Raji group (Fig. 3b, c). However, the proportions of T cells in mouse peripheral blood and spleen in Raji and Raji-MIP3α groups exhibited no significant differences at day 3 and 5 (*P* > 0.05; supplementary Fig. 6d, e), implying that MIP3α overexpressed in tumors could induce directed migration of T cells into tumor tissues. We performed IHC to further confirm the T-cell accumulation in tumor tissues overexpressing MIP3α. Consistently, the experimental data showed that tumor-infiltrating T cells in Raji-MIP3α tumors were substantially higher than in Raji tumors at day 5 (average density: 3.36 vs. 0.91, *P* = 0.003, Fig. 3d-e). To evaluate the anti-tumor effect of CAR-T cells, we dynamically monitored the tumor volume of mice till the 31st day after CAR T-cell infusion (Fig. 3a). Our results showed that the tumor size of mice in the Raji-MIP3α group was significantly smaller than that in Raji group (*P* = 0.0078, Fig. 3f-g), and no significant difference was seen in the body weight between mice engrafted with Raji or Raji-MIP3α cells (*P* = 0.8803, supplementary Fig. 6f). The results showed that tumorous regions with elevated MIP3α expression could increase the infiltration of T lymphocytes, thereby enhancing the anti-tumor efficacy of CAR-T therapy.

## Discussion

While unprecedented progress has been made in CAR-T therapy recently, long-term remission has been hindered by antigen-negative relapse. Our center was the first set to employ sequential infusion of CAR19/22 T cells for the treatment of B-cell malignancies (ChiCTR-OPN-16008526), with promising clinical efficacy accomplished [2]. In our study, we retrospectively analyzed the recurrence pattern in these patients and results showed that most of B-ALL patients (96%) and half of B-NHL patients attained CR. Target-negative relapse rates were significantly lowered in our cohort. However, some patients still experienced relapse later. Our study found that most patients developed recurrence within 6 months of sequential CAR T-cell infusion, and patients attaining remission for over 6 months were less likely to relapse and achieved prolonged survival. The analysis of the recurrence signature of CAR19/22 T-cell therapy is new and clinically relevant. Given that most recurrences occurred in the early stages of CAR T-cell infusion and salvage regimens after recurrence rarely accomplished long-term remissions, early identification of patients at higher risk of NR/ER after CAR T-cell infusion was significant in the decision-making of appropriate intervention following CAR T-cell treatment. However, the potential biomarkers for sequential CAR19/22 T-cell therapy are not available so far. In this study, MIP3α was found to have relatively higher predictive power for identifying patients with NR/ER. In these patients, the detection of the predictive cytokines can allow for adequate time to bridge to other treatments. MIP3α, with relatively balanced specificity and sensitivity, can serve as a promising prognostic biomarker for patients after sequential CAR T-cell infusion.

**Fig. 4:**
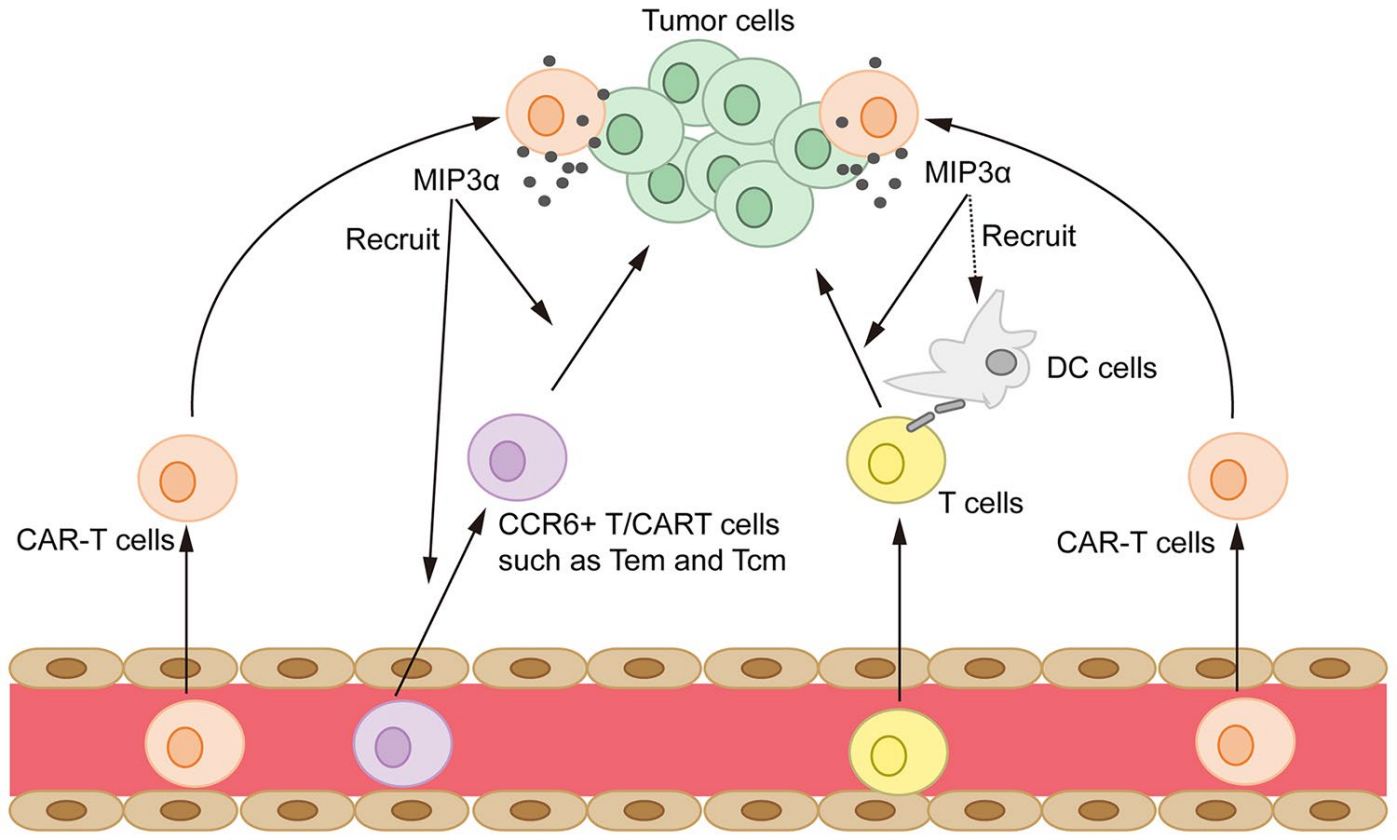
A scheme of the proposed mechanism of the MIP3α-mediated superior efficacy of CAR T cells Well-responding CAR-T cells are activated upon exposure to tumor cells, thereby promoting MIP3α secretion. Elevated MIP3α recruits more T cells, especially T lymphocytes with the memory phenotype (CCR6-positive), to the tumor site. Prior studies also suggested that MIP3α could recruit DCs to activate endogenous T cells in tumors (dashed arrows). This may explain why MIP3α can serve as a prognostic predictor with high sensitivity and specificity for identifying patients at higher risk for NR/ER at an earlier time point

So far, multiple studies tried to identify factors that were related to the clinical response to CAR-T therapy and could serve as indicators for clinical management. Apart from tumor burden, the composition of the tumor microenvironment (TME) and the functional status of CAR-T cells are hot spots of research. The former includes suppressive immune cells like MDSC [21] and TAM [20] and cytokines/chemokines such as MCP-1 and IL-7 [13], and the latter involves immuno-phenotypes of CAR-T cells, such as the percentage of less differentiated T cell subsets [12] and the persistence of CAR-T cells in vivo [15]. MIP3α, uncovered by our study, might reflect both TME and the status of CAR-T cells. The HPA database indicates that MIP3α is mainly derived from MAIT and memory T cells (*proteinatlas.org*). A previous study showed that CAR-T cells stimulated by target cells up-regulated the expression of genes in JAK/STAT3 signaling pathways, including MIP3α, which was consistent with our ELISA data. Moreover, the elevated level of MIP3α in CR patients was higher than in PR/NR patients, suggesting that CAR-T cells in well-responding patients might express more MIP3α [12]. Our data also showed that MIP3α levels at baseline were similar across patients and much lower than those at the peak of CRS. Therefore, we are led to speculate that baseline MIP3α levels in lymphoma tissue before CAR T-cell infusion would be very low. In the future, the availability of tumor tissues prior to CAR-T infusion would further confirm the source of MIP3α. On the other hand, the role of the MIP3α-CCR6 axis was reported to attract dendritic cells (DC), effector/memory T cells and B cells, thereby taking part in the pathogenesis of inflammatory and infectious diseases and several malignancies [22]. The chemotactic effect of MIP3α has been confirmed in the chemotaxis assays and animal experiments also showed that tumors overexpressing MIP3α could chemoattract more T cells into the tumor environment, which further enhanced the tumoricidal effect of CAR-T cells. These findings might explain the close correlation between MIP3α level and the antitumor activity of CAR T-cell therapy (Fig. 4).

High-level inflammatory cytokines also have been used for the prediction of CAR-T efficacy and some of them appeared to be associated with the onset of CRS [23]. Up till now, the correlation between CRS and CAR T-cell effects remained controversial and the severity of CRS was affected by many factors, including patient-specific factors such as tumor burden [3] and treatment-related factors such as doses of CAR-T cells and the administration of tocilizumab [24]. As shown in supplementary Fig. 3l, the Kaplan–Meier curves in B-NHL patients with grade 3–5 CRS differed from those with grade 0–2 CRS, although the difference was not statistically significant (*P* = 0.0954). This finding suggested that CRS severity might partially reflect the response of CAR T-cells, but did not suffice to act as a prognostic indicator. In our study, MIP3α, as a promising prognostic biomarker for patients following CAR19/22 cocktail CAR T-cell therapy, was not affected by CRS severity. It might be more reliable as a prognostic biomarker.

The issue of poor CAR T-cell trafficking is important to CAR-T treatment, not only to solid tumors but also to hematologic malignancies. A previous study showed that B-NHL patients in complete remission had significantly increased T/CAR-T cells, and high infiltration of TAM, suppressing T-cell proliferation, was negatively correlated with remission status [20]. CAR-T cells in tandem with IL-7 and CCL19 reportedly increased DC and T-cell infiltration and prolonged CAR T-cell survival [25]. Similarly, our results indicated that MIP3α could recruit more immune cells, such as T lymphocytes, into the tumor tissues. Increasing tumor-infiltrating immune cells is known to be associated with more favorable clinical outcomes of CAR-T treatment. Therefore, MIP3α, which could attract T cells without affecting the tumoricidal effect of CAR-T therapy, has the potential to be used with CAR-T cells to improve the efficacy, through CAR-T cells armored with MIP3α. CAR-T cells expressing relevant chemokine receptors such as CCR4, CXCR1 or CXCR2 have been reported to enhance the efficacy of immunotherapy [26–29]. Similar to these studies, CAR-T cells overexpressing CCR6, the receptor of MIP3α, might be effective in poorly responsive patients with high baseline serum MIP3α levels in tumor tissues. In the future, the specific efficacy of CCR6-expressing CAR-T cells could be further investigated after the identification of CAR-T-resistant tumors with high levels of baseline MIP3α.

In summary, this study showed that relapse occurred mainly within six months after sequential CAR19/22 T-cell infusion. Moreover, MIP3α could act as a valuable post-infusion biomarker for identifying patients with NR/ER, and could contribute to the early intervention after CAR-T therapy. By taking advantage of the synergistic effect of MIP3α, CAR-T cells might be modified to further improve the efficacy of cellular immunotherapy. More investigations are needed to identify factors that dictate the clinical outcomes of CAR-T treatment to achieve long-term remission for most patients.

## Supplementary Information

Below is the link to the electronic supplementary material.

Supplementary file1 (DOCX 2323 KB)

Supplementary file2 (XLSX 71 KB)

## Abbreviations

AUC: Area under each ROC curve
B-ALL: B-cell acute lymphoblastic leukemia
B-NHL: B-cell non-Hodgkin lymphoma
CAR-T: Chimeric antigen receptor-T
CAR19/22: Anti-CD19 and anti-CD22 CAR
CCR6: C–C chemokine receptor 6
CI: Confidence interval
CM: Central memory
CR: Complete response
CRS: Cytokine release syndrome
DC: Dendritic cells
EM: Effector memory
ER: Early relapse
EGFRt: Truncated epidermal growth factor receptor
FISH: Fluorescence *in-situ* hybridization
GMP: Good Manufacturing Practice
GVHD: Graft-*versus*-host disease
HSCT: Hematopoietic stem cell transplantation
IFN‐γ: Interferon‐γ
IHC: Immunohistochemistry
IL: Interleukin
IOD: Integrated optical density
MIP3α: Macrophage inflammatory protein-3α
MOI: Multiplicity of infection
MRD: Minimal residual disease
NCCN: National Comprehensive Cancer Network
NR: Non-response
OS: Overall survival
PBMCs: Peripheral blood mononuclear cells
PET/CT: Positron emission tomography/computed tomography
PFS: Progression-free survival
PR: Partially responding
ROC: Receiver operating characteristic
scFv: Single-chain variable fragments
TME: Tumor microenvironment
TNFα: Tumor necrosis factor‐α
