# Supplementary material for: MIP3α as an early prognostic predictor for patients with B-cell malignancies receiving CD19/CD22-redirected CAR-T cell cocktail therapy
Source: Cancer Immunol Immunother. 2023 Mar 4;72(7):2245–56. doi: 10.1007/s00262-023-03418-2 (PMC10264474; doi:10.1007/s00262-023-03418-2)
Supplement: Supplementary file 1 — Supplementary file1 (DOCX 2323 KB) [file 262_2023_3418_MOESM1_ESM.docx]

# *Supplementary Material*

# 1 Supplementary Methods

## Patients and CAR-T regimen

Between October 2017 and August 2019, we recruited 119 consecutive patients with aggressive B-cell malignancies who had been involved in the clinical trial of sequential infusion of anti-CD19 and anti-CD22 CAR-T therapy (ChiCTR-OPN-16008526 at [*http://www.chictr.org.cn*](http://www.chictr.org.cn)) (1). The patients in our cohort and subjects in wang et al.’s cohort (1) overlapped from October 2017 to January 2018, including 9 B-ALL patients and 16 B-NHL patients. Before CAR-T treatment, target antigen expression on tumor biopsy samples was detected by flow cytometry or immunohistochemistry (IHC), and CD19 and CD22 antigens on tumor B cells were found to be highly expressed in all subjects enrolled (>90%). The lymphodepletion chemotherapy consisted of fludarabine 25 mg/m^2^ and cyclophosphamide 300 mg/m^2^ for three days (days -4 to day -2), administered before CAR-T cell infusion. And then, CAR22 and CAR19 T cells were separately divided into two equal doses and sequentially infused on successive days from day zero. CAR22 T cells were consistently infused first and the entire infusion process lasted for about four days in a row. The target doses for patients with B-ALL and B-NHL were 2.5×10^^6^/kg and 5.0×10^^6^/kg, respectively (supplementary Fig. 1). Clinically, however, for patients over 65 years old or suffering from serious comorbidity or high disease burden, CAR19 and CAR22 T cells were infused with escalating doses. Therefore, the actual doses of CAR-T cells in B-ALL and B-NHL patients would be marginally higher than the target doses. This study was conducted upon approval by the Medical Ethics Committee of Tongji Hospital, Tongji Medical College, Huazhong University of Science and Technology, Wuhan, China, and all patients provided informed consent in strict accordance with the Declaration of Helsinki.

## Treatment response and adverse events

Efficacy was evaluated on monthly basis for half a year and then every 3 months, in accordance with the National Comprehensive Cancer Network (NCCN) guidelines and Lugano Treatment Response Criteria (2). All patients were followed up until transplantation, death, loss to follow-up or cutoff date for data collection (30 August 2020). The evaluation and management of cytokine release syndrome (CRS) or other adverse events were based on the CRS criteria proposed by Lee et al (3) and the National Cancer Institute Common Terminology Criteria for Adverse Events v.5.0 (4). Minimal residual disease (MRD) screening was performed by employing positron emission tomography/computed tomography (PET/CT) or CT and flow cytometry. Genomic aberrations were identified by fluorescence *in-situ* hybridization (FISH) and next-generation exome sequencing.

## Cell lines and cell culture

Burkitt lymphoma cell line Raji and erythroleukemia cell line K562 were cultured in RPMI 1640 medium (Gibco, Grand Island, NY, USA) supplemented with 10% fetal bovine serum (FBS; Gibco, Grand Island, NY, USA). The lentivirus packaging cell line LentiX™293T (Takara Biomedical Technology, Beijing, China) was cultured in DMEM medium (Gibco, Grand Island, NY, USA) containing 10% FBS. All cell lines were preserved in and obtained from our laboratory and verified before use. Raji‐MIP3α, a human MIP3α‐expressing Raji cell line, was constructed by transfection with the lentivirus containing MIP3α expression vectors.

## Recombinant plasmid construction and lentivirus packaging

The clinical trial used the CAR consisting of an anti-CD19 or CD22 scFv (single-chain variable fragments), a CD8a hinge/transmembrane region, the CD28 and 4-1BB costimulatory domains, and intracellular CD3ζ. The anti-CD19 and -CD22 scFvs were derived from the FMC63 clone under patent WO2012079000 and the m971 clone under patent US8591889B2, respectively (1). For laboratory verification, the CD19 CAR gene was linked to a truncated epidermal growth factor receptor (EGFRt) by T2A sequence to facilitate *in vitro* detection. The CAR19 gene and the MIP3α gene were connected through IRES sequence to form the CAR19-MIP3α plasmid. Lenti-X™293T cells were co-transfected with the expression vectors with psPAX2 and PMD2.G packaging plasmids. The viral supernatants were collected, filtered, and concentrated 72 h after transfection, and then aliquoted and stored at -80 °C. For virus titration, 293T cells (3×10^^5^/ml) were seeded into a 96-well plate one day in advance. After adhesion, 2 μl of the cryopreserved purified virus solution was added to 198 μl of complete medium. And then 100 μl of the aforementioned liquid was taken for repeated two-fold dilution to obtain six concentration gradients. The diluted virus suspension was added to the 96-well plate to allow for interaction with 293T cells. After 48 hours, the positive rate of EGFR in each well was detected by flow cytometry. Lentiviral titer (TU/ml) = (cell number in each well × EGFR positive rate (%) ×10)/virus loading volume in each well (μl). Recombinant plasmids aforementioned were extracted by using the EndoFree Plasmid Maxi Kit (Qiagen, Hilden, Germany) according to the manufacturer’s instructions.

## Isolation, activation, transduction and culture of T cells

Peripheral blood mononuclear cells (PBMCs) were isolated by density gradient centrifugation on Ficoll‐Paque Plus (GE Healthcare, Boston, MA, USA). CD3+ T cells from PBMCs were separated using CD3 microbeads (Miltenyi Biotec, Bergisch Gladbach, Germany) by following the manufacturer’s instructions. Then, T cells were activated with Dynabeads™ Human T-Activator CD3/CD28 (Gibco, Grand Island, NY, USA) in CTS™ OpTmizer™ medium (Gibco, Grand Island, NY, USA) supplemented with 5% human AB serum, 2 mM l-glutamine (Gibco, Grand Island, NY, USA) and 200 IU/mL IL‐2 (PeproTech, Rocky Hill, NJ, USA). After 1 day of stimulation, primary human T cells were transduced with concentrated lentivirus at a multiplicity of infection (MOI) ranging from 2 to 5. Twenty-four hours later, the T cells were centrifuged and resuspended in fresh culture medium at a density of 1-2×10^^6^/ml. Functional assays were performed after 14 days of *in vitro* culture. The manufacture of CAR-T cells strictly followed the Good Manufacturing Practice (GMP). Quality control of CAR-T products for each patient was exercised and CAR-T cells were administered strictly against the release criteria, including (1) Transfection efficiency and CAR expression in CAR-T products; (2) Tumor-killing efficiency of CAR-T cells; (3) cell counts and viability; (4) Sterility testing; (5) endotoxin level; (6) residual Dynabeads™ T-activator CD3/CD28.

## Flow cytometry

For CAR detection, CAR‐T/T cells were stained with AF488 or APC‐conjugated mouse anti‐human EGFR antibody (clone: AY13; BioLegend, San Diego, CA, USA) or PE-labeled human CD19 protein (ACROBiosystems, Beijing, China, Cat. CD9-HP2H3). To determine the expression level of C-C chemokine receptor 6 (CCR6), PBMCs were stained with BB515-conjugated anti-CCR6 (clone: 11A9, BD Pharmingen, Franklin Lakes, NJ, USA), APC-CY7-conjugated anti-CD45 (clone: HI30, Agilent, Santa Clara, Calif, USA), PerCP-conjugated anti-CD3 (clone: SK7, BDIS, Franklin Lakes, NJ, USA), PE-conjugated anti-CD45RO (clone: UCHL1, BDIS, Franklin Lakes, NJ, USA), BV605-conjugated anti-CD62L (clone: DREG-56, BD Pharmingen, Franklin Lakes, NJ, USA), APC-conjugated anti-CD14 (clone: MfP9, BDIS, Franklin Lakes, NJ, USA), BV421-conjugated anti-CD19 (clone: HIB19, BioLegend, San Diego, CA, USA), BV510-conjugated anti-CD56 (clone: HCD56, BioLegend, San Diego, CA, USA) antibodies. To analyze the effect of MIP3α on T-cell subsets, CAR‐T/T cells on day 7, 10, 13 and 16 of *in vitro* culture were stained with APC-conjugated anti-CD4 (clone: OKT4, BioLegend, San Diego, CA, USA), PE/cyanine7-conjugated anti-CD8 (clone: SK1, Agilent, Santa Clara, Calif, USA), PE-conjugated anti-CD45RO and BV605-conjugated anti-CD62L antibodies. More details are given in supplementary Table 1. Data acquisition was conducted on a NOVOCYTE-D3000 instrument.

## Cell migration assay

Migration of the PBMCs was evaluated through a permeable polycarbonate filter in 24-well transwell chambers (Corning, NY, USA; 6.5 mm diameter, 5.0-µm pore size). The PBMCs (2×10^^6^/ml) were placed in the upper chambers, and the lower chambers were loaded with recombinant MIP3α (PeproTech, Rocky Hill, NJ, USA; 1 μg/ml) or RPMI 1640 medium alone. After 4 hours of incubation, cells in the bottom chambers were harvested together with 5×10^^4^ LentiX™293T cells for quantification and to reduce centrifugation losses. All cells were stained with Pacific Blue-conjugated anti-CD3 (clone: SK7, BioLegend, San Diego, CA, USA), BV785-conjugated anti-CCR6 (clone: G034E3, BioLegend, San Diego, CA, USA), BB515-conjugated anti-CD19 (clone: HIB19, BD Pharmingen, Franklin Lakes, NJ, USA), PE-CY7-conjugated anti-CD56 (clone: NCAM16.2, BDIS, Franklin Lakes, NJ, USA), PE-conjugated anti-CD45RO and BV605-conjugated anti-CD62L antibodies for flow cytometrical analysis.

## Detection of cytokine production

The expression levels of MIP3α in primary T cells and cell lines were detected by using human MIP-3 alpha ELISA kit (RayBio®, Norcross, GA, USA, Cat. ELH-MIP3a). Cytokine-releasing values of CAR‐T/T cells were measured by using human IL-2, tumor necrosis factor‐α (TNFα), interferon (IFN)‐γ and Granzyme B ELISA kit (NEOBIOSCIENCE, China) by following the manufacturer’s instructions. Effector cells (control T, CAR- or CAR-MIP3α T cells) were co-incubated with target cells (Raji or K562) at an E: T ratio of 1:1 in 96-well plates for 24 h and 48 h. Culture supernatants were collected for ELISA.

## *In vitro* cytotoxicity

To determine the cytotoxicity of CAR-T cells, target cells (Raji, K562) were labeled with calcein (Aladdin, Shanghai, China) and then seeded into each well of a 96‐well plate in triplicate (5×10^^4^/ml), with effector cells (control T, CAR- or CAR-MIP3α T cells) at specified effector‐to‐target (E: T) ratios (9:1, 3:1 and 1:1). The plate was centrifuged after 4 hours of incubation, and the supernatants were then transferred into another black 96-well plate. Wells with only target cells were considered to be spontaneous release wells, and wells with target cells and lysis solution served as maximum release wells. The fluorescence of each well was detected on a multifunctional microplate reader.

## CD107a assay

For degranulation assay, effector cells (control T, CAR- or CAR-MIP3α T cells) were co-incubated with target cells (Raji or K562) at an E: T ratio of 1:1 in the presence of 1:50 APC-conjugated anti-CD107a antibody (clone: H4A3, BioLegend, San Diego, CA, USA) and 1:500 monensin (Golgi-Stop, BioLegend, San Diego, CA, USA) for about 4 hours. The cells were then stained with PerCP-conjugated anti-CD8 (clone: SK1, BDIS, Franklin Lakes, NJ, USA) and AF488-conjugated anti-EGFR (clone: AY13, BioLegend, San Diego, CA, USA) antibodies for the detection of CD107a expression.

## IHC

Formalin-fixed, paraffin-embedded slices were baked at 65 °C for 2 hours, deparaffinized twice in xylene, and then rehydrated through graded alcohol. To retrieve antigenicity, slices were heated in a microwave oven (medium fire) in boiling citrate acid repair buffer for 25-30 min and then cooled naturally. The slices were incubated in 3% hydrogen peroxide for 30 min at room temperature to block endogenous peroxidase. Afterward, slices were incubated with 3% BSA for 60 min. Subsequently, slices were placed in a humidified chamber at 4 °C overnight with the primary antibodies specific for CD3 (dilution 1:150; Abcam, ab1666). On the second day, slices were washed 3 times and incubated with the secondary antibody (HRP enzyme label) corresponding to the primary antibody for 60 min, protected from light at room temperature. Then, slices were stained with DAB substrate, counterstained with hematoxylin and differentiated with 1% hydrochloric acid and alcohol. Finally, slices were sealed with neutral gum and photographed on a microscope (Olympus). The positive staining region was quantitatively analyzed by using Image-Pro Plus. The average density was calculated as follows: Average density = Integrated optical density (IOD) / Area of interest.

## *In vivo* antitumor effect

Thirty 6-week-old female NCG mice (GemPharmatech Co., Ltd., Nanjing, China) were randomly engrafted with 5×10^^6^ Raji or Raji-MIP3α cells (resuspended in 100 μl of sterile PBS) onto the right side of the back by subcutaneous injection. Twelve days later, 2.5×10^^6^ CAR-T cells were infused into the mice with tumor volumes of about 100-400 mm^3^ via tail injection (mice with oversized or undersized subcutaneous tumors were excluded, n = 12 in each group; see supplementary Fig. 2). Body mass and tumor burden were regularly measured with a caliper twice a week by the same experimenters, and the tumor volume was calculated as follows: V = (tumor width) ^^2^ × tumor length / 2. Three and five days after CAR T-cell infusion, 4 mice from each group were randomly taken and euthanized and tumors were resected. Half of the tumors were digested according to the manufacturer’s instructions (Miltenyi Biotec, Bergisch Gladbach, Germany) and the tumor-infiltrating T cells were detected by flow cytometry with V500-conjugated anti-CD45 (clone: HI30, BD Pharmingen, Franklin Lakes, NJ, USA), Pacific Blue-conjugated anti-CD3 (clone: SK7, BioLegend, San Diego, CA, USA) and PE-conjugated anti-CD19 (clone: HIB19, BioLegend, San Diego, CA, USA) antibodies. Meanwhile, T cells from peripheral blood and spleen were analyzed by FACS. The remaining resected tumors were fixed with 4% paraformaldehyde and embedded in paraffin for IHC. The average density was calculated as follows: Average density = Integrated optical density (IOD) / Area of interest. The experimenter after CAR T-cell infusion was unaware of the tumor cells engrafted with the mice, and data points were considered outliers if they exceeded the mean ± 3-fold standard deviations. All animal experiments were approved by the Institutional Committee of Animal Care of Tongji Hospital, Tongji Medical College, Huazhong University of Science and Technology, Wuhan, China. All experiments were performed under specific pathogen-free conditions at the animal experimental center of Tongji Hospital.

## Statistical analysis

SPSS 25.0 software package was used for data analysis. Unless otherwise stated, all the data were representative of the results of at least three independent experiments. PFS and overall survival (OS) were evaluated as the time from CAR T-cell infusion to the first recurrence or death, respectively. The survival was presented by using Kaplan-Meier curves and compared by log-rank test. The 95% confidence interval [CI] was measured by the Clopper-Pearson method. Receiver operating characteristic (ROC) curves (5), a plot of the sensitivity versus (1-specificity) of a diagnostic test, were used to assess the predictive power of biomarkers. *Youden*'s index (6) equals sensitivity + (1-specificity) and was used to obtain the optimal cutoff value of a biomarker that yielded maximal sensitivity and specificity. The normality test was first performed on the experimental data. Two-tailed unpaired t-test, one-way or two-way analysis of variance, and non-parametric test were employed to compare quantitative data between groups. A *P* value <.05 was considered to be statistically significant. Graphs were drawn by using GraphPad Prism software v.9.0.0, and figures were formatted using Adobe Illustrator CS6.

# 2 Supplementary Figures

## 2.1 Supplementary Fig. 1

**
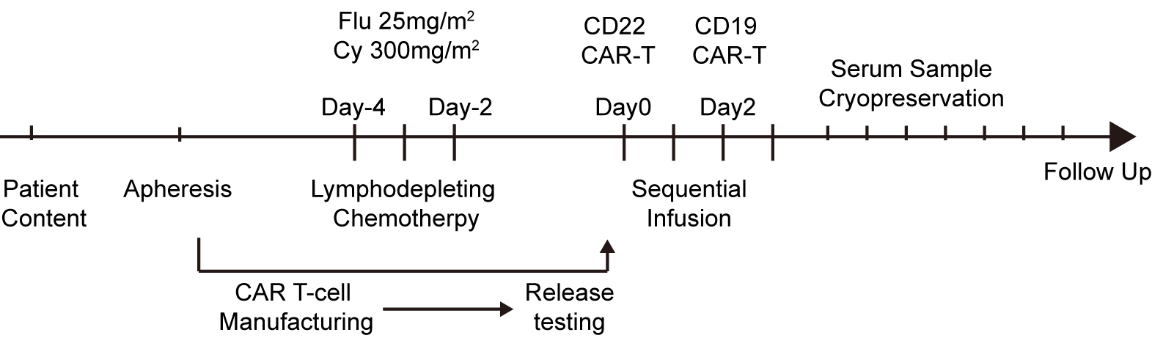
**

**Supplementary Fig. 1 Schematic diagram of the clinical protocol.** Before CAR-T treatment, target antigen expression on tumor biopsies was detected by flow cytometry or IHC. Patients were subjected to lymphodepletion chemotherapy composed of fludarabine 25 mg/m^2^ and cyclophosphamide 300 mg/m^2^ for 3 days (days -4 to day -2). CAR22 and CAR19 T cells were performed quality control assays and then separately divided into two equal doses and sequential infusion on successive days from day zero. CAR22 T cells were always infused firstly. In our cohort, no patients received only one type of CAR-T cells. The target dosing for patients with B-ALL and B-NHL were 2.5×10^^6^/kg and 5.0×10^^6^/kg, respectively. Serum samples were collected during the first 30 days after sequential CAR T-cell infusion. Efficacy evaluation was conducted monthly for half a year and then every 3 months.

## 2.2 Supplementary Fig. 2

**
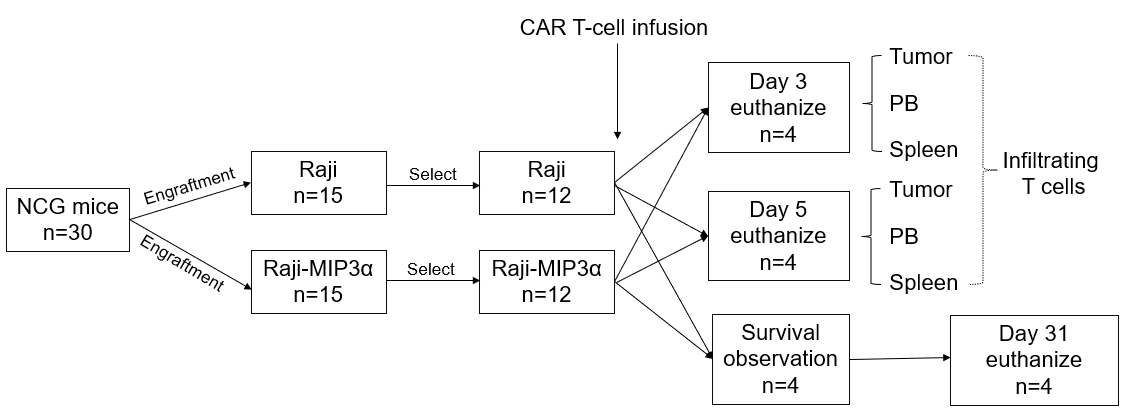
**

**Supplementary Fig. 2 The graphical representation of the animal experiments.** The numbers of experimental mice were detailly shown. Thirty 6-week-old female NCG mice were randomly engrafted with 5×10^^6^ Raji or Raji-MIP3α cells by subcutaneous injection. Twelve days later, 2.5×10^^6^ CAR-T cells were infused into the mice with tumor volumes between 100-400 mm^3^ via tail injection (mice with oversized or undersized subcutaneous tumors were excluded, n = 12 in each group). Body mass and tumor burden were regularly measured twice a week. Three and five days after CAR T-cell infusion, 4 mice from each group were randomly grabbed and euthanized and tumors were resected for flow cytometry and IHC analysis. The remaining mice were monitored until 31 days after CAR T-cell infusion.

## 2.3 Supplementary Fig. 3


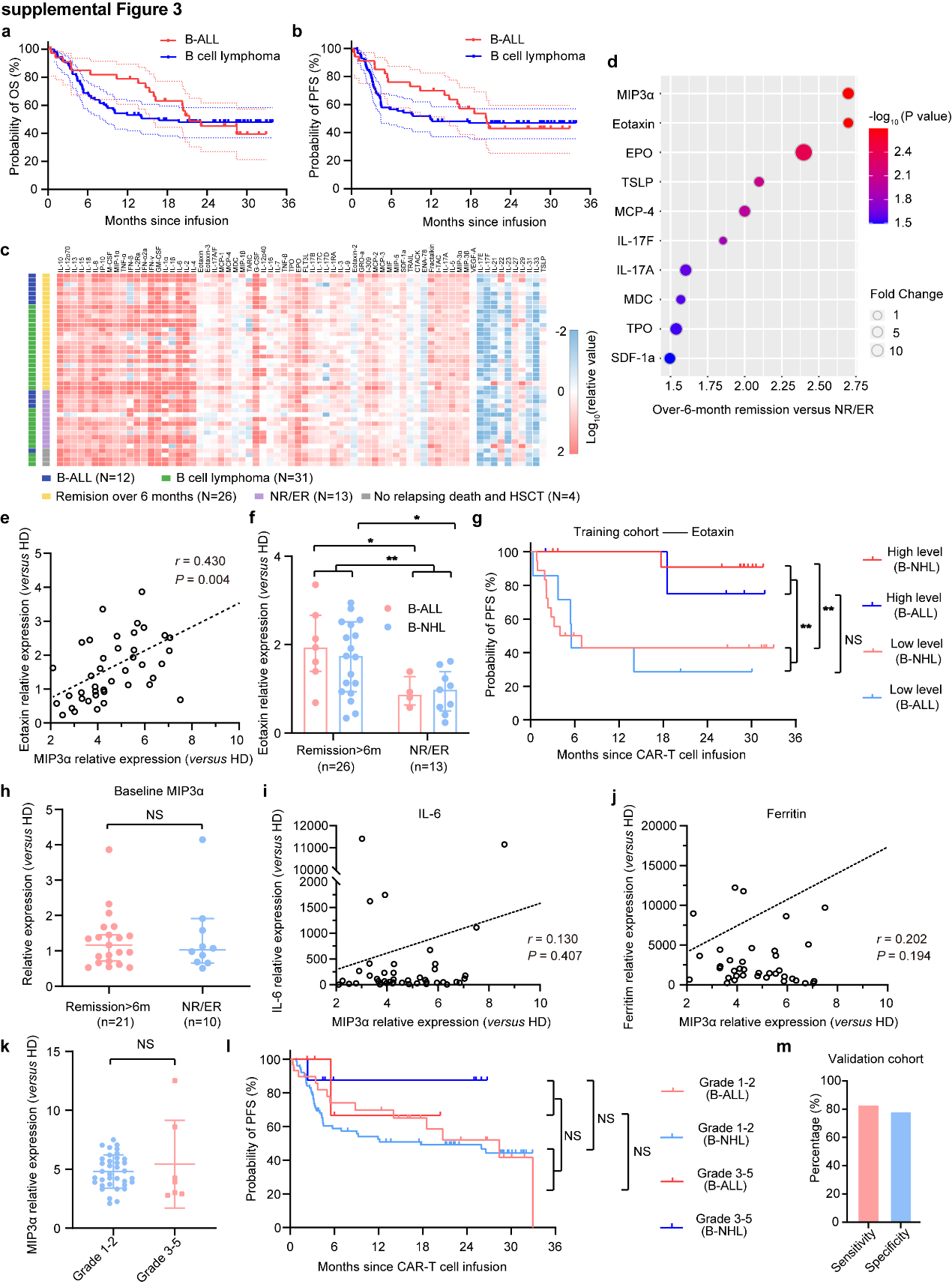


**Supplementary Fig. 3 Screening for cytokines associated with prognosis.** OS (a) and PFS (b) in this cohort, including B-ALL and B-NHL patients. (c) Relative values of 70 biomarkers (*versus* healthy donors) are presented in a heatmap. (d) Dot plot illustrating *P* values and fold changes (*versus* healthy donors) of cytokines with significantly different. (e) The levels of Eotaxin correlated with the levels of MIP3α (r = 0.430; *P* = 0.004). (f) Eotaxin showed statistical differences in serum levels between patients with NR/ER and those with over-6-month remission in the training group (*P* = 0.0018). (g) The curve of PFS according to Eotaxin levels in the training group. (h) Baseline MIP3α showed no statistical differences between patients with NR/ER and those with over-6-month remission. The expression of MIP3α was not correlated with the levels of IL-6 (i) and Ferritin (j). (k) MIP3α showed no statistical differences in serum levels between patients with grade 1-2 CRS and those with grade 3-5 CRS (*P* = 0.551). (l) The efficacy of CAR-T therapy was not associated with CRS. (m) The sensitivity and specificity of MIP3α in the validation group.

## 2.4 Supplementary Fig. 4


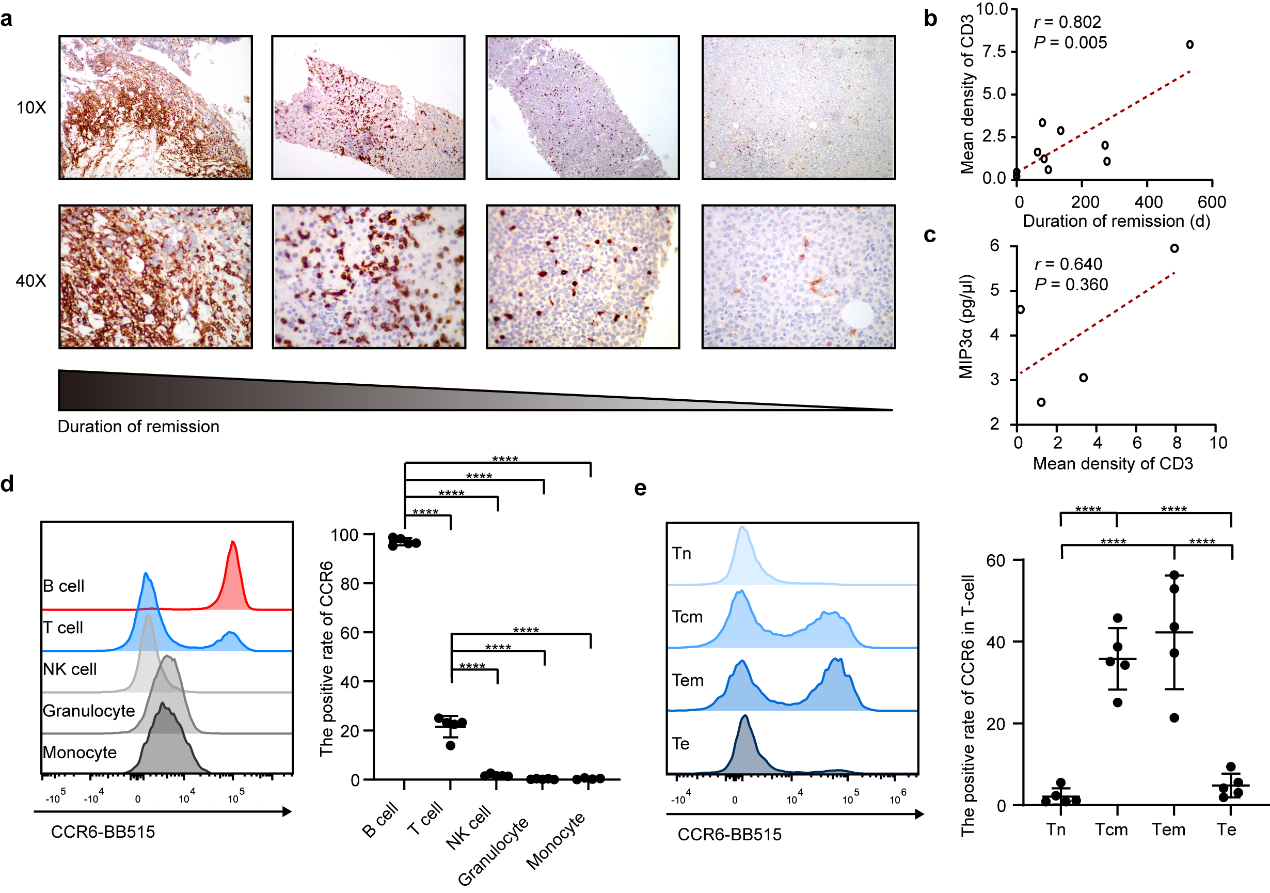


**Supplementary Fig. 4 Patients with less T-cell infiltration might have lower serum MIP3α levels.** (a) IHC staining of CD3 expression in tumor samples collected from B-NHL patients at the time points of relapse. The average density was analyzed from 3 randomly selected regions. (b) The duration of remission was correlated with the level of MIP3α (r = 0.802; P = 0.005). (c) The expression of MIP3α was positively correlated with CD3 expression in tumor samples (r = 0.640). (d) Expression levels of CCR6 in PBMCs, including B cells, T cells, NK cells, granulocytes and monocytes. Data represent the average from five donors. (e) Expression levels of CCR6 in memory-phenotype T subsets. Data represent the average from five donors.

## 2.5 Supplementary Fig. 5


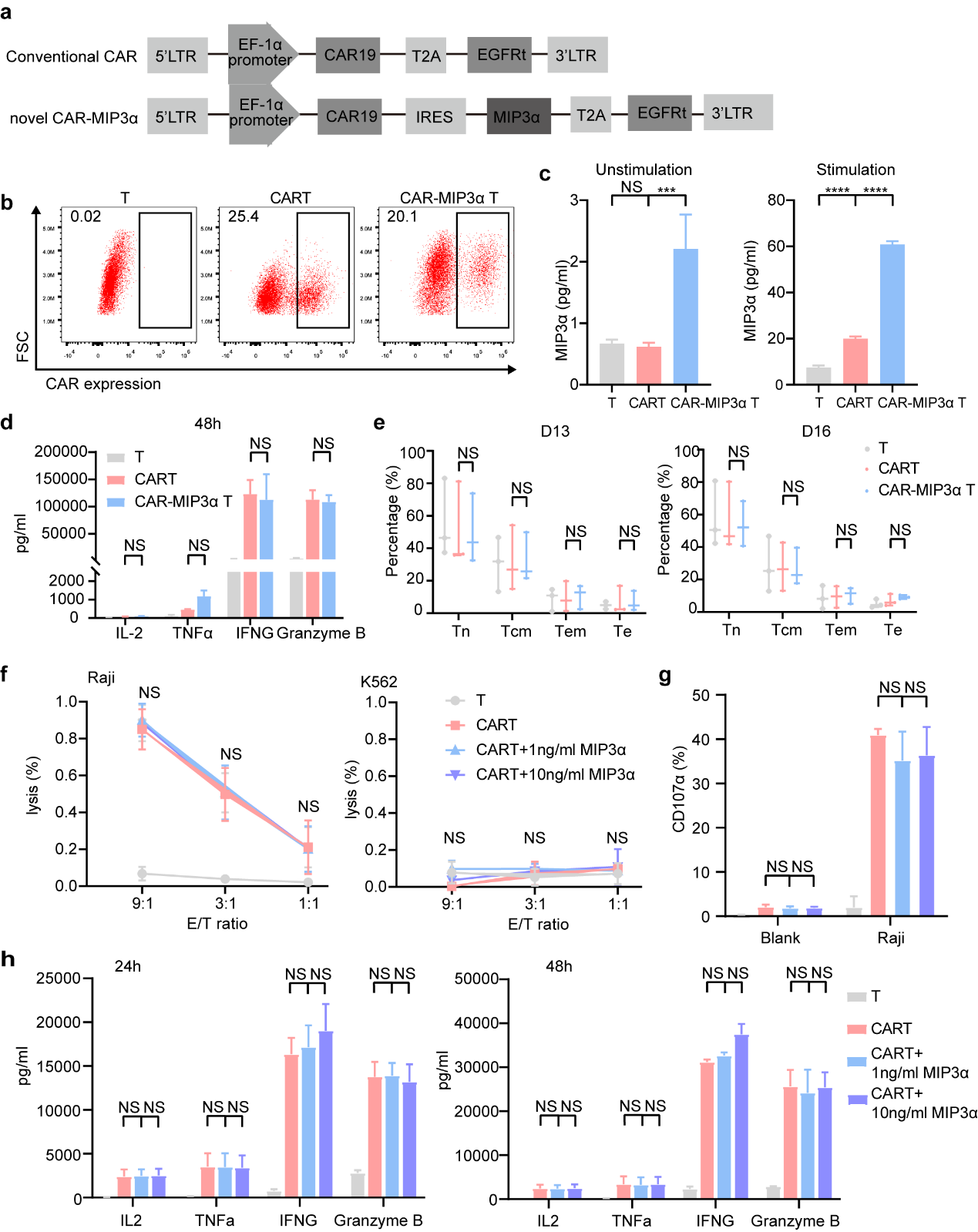


**Supplementary Fig. 5 MIP3α exerted no influence on anti-tumor effects of CAR-T cells.** (a) CAR-vector construction strategy. The CAR19 gene and the MIP3α gene were connected through IRES sequence to form the CAR-MIP3α plasmid. (b) Transfection rate of CAR- and CAR-MIP3α T cells. (c) The secretion of MIP3α by T, CAR-, CAR-MIP3α T cells in resting and stimulation states. (d) Quantification of cytokines (IL-2, TNFα, IFN‐γ and Granzyme B) from the supernatant after CAR-T/T cells co-cultured with Raji at an E: T ratio of 1:1 for 48 h. (e) Frequency of CM, EM, effector and naïve T cells in CAR-T/T cells as assessed by using flow cytometry on day 13 and 16. The cytotoxicity (f), degranulating effect (g) and cytokines secretion (h) of T, CAR-T cells and CAR-T cells supplemented with different concentrations of MIP3α were determined after incubation with Raji cells. Two-way ANOVA was conducted for statistical analysis. NS: *P* > 0.05, *** *P* < 0.001 and **** *P* < 0.0001.

## 2.6 Supplementary Fig. 6


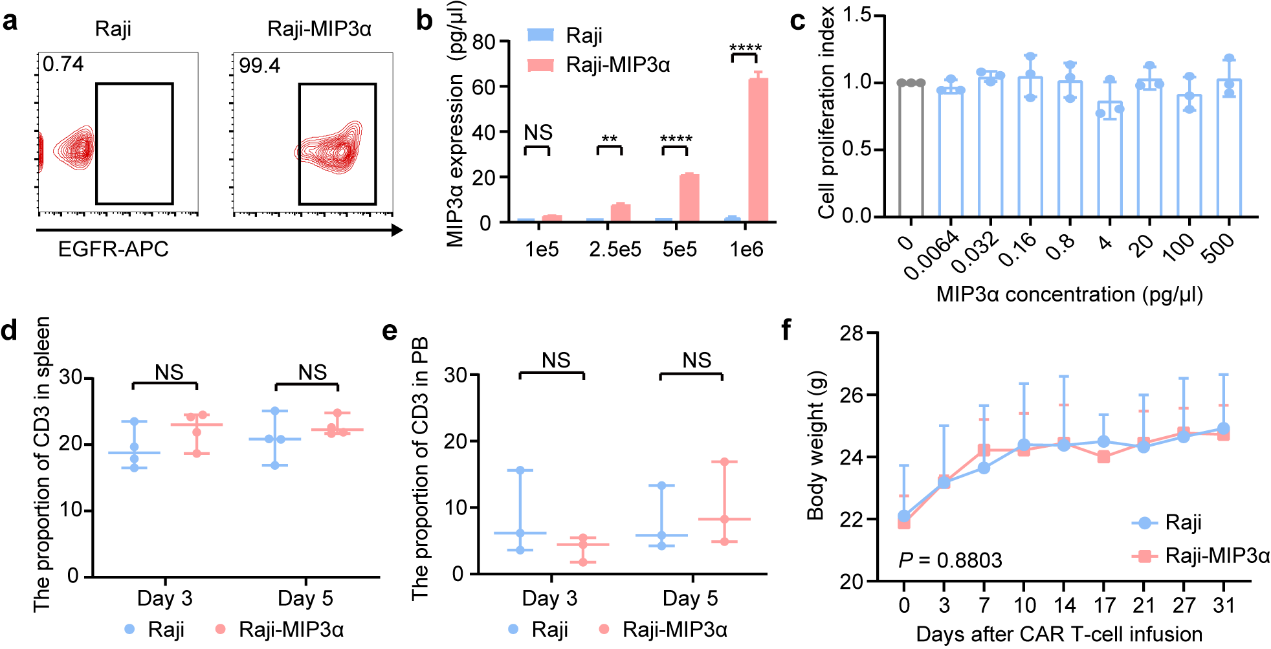


**Supplementary Fig. 6 MIP3α improved the therapeutic effect of CAR-T cells in vivo.** (a) The MIP3α gene expression was detected by flow cytometry in Raji- MIP3α cells. (b) the secretion of MIP3α was quantified by ELISA at 24 h. (c) MIP3α had no effect on the proliferation of target cells Raji by using CCK8 methods. The proportion of T cells in the spleen (d) and peripheral blood (e) were evaluated by flow cytometry. (f) The body mass of mice was assessed after CAR T-cell infusion. Two-way ANOVA was conducted for statistical analysis. NS: *P* > 0.05, *****P* < 0.0001.

| 3 Supplementary Tables3.1 Supplementary Table 1  \| **Supplementary Table 1. Antibodies for flow cytometry** \| \| \| \| \| \| \| --- \| --- \| --- \| --- \| --- \| --- \| \| **Fluorescence** \| **Type** \| **Clone** \| **Supplier** \| **Country** \| **Cat#** \| \| AF488 \| anti-human EGFR \| AY13 \| BioLengend \| San Diego, CA, USA \| 352908 \| \| APC \| anti-human EGFR \| AY13 \| BioLengend \| San Diego, CA, USA \| 352906 \| \| PE \| CD19 protein \| - \| ACROBiosystems \| Beijing, China \| CD9-HP2H3 \| \| BB515 \| anti-human CCR6 \| 11A9 \| BD Pharmingen \| Franklin Lakes, NJ, USA \| 564479 \| \| BV785 \| anti-human CCR6 \| G034E3 \| BioLengend \| San Diego, CA, USA \| 353421 \| \| APC-CY7 \| anti-human CD45 \| HI30 \| Agilent \| Santa Clara, Calif, USA \| 8930264 \| \| PerCP \| anti-human CD3 \| SK7 \| BDIS \| Franklin Lakes, NJ, USA \| 652831 \| \| PE \| anti-human CD45RO \| UCHL1 \| BDIS \| Franklin Lakes, NJ, USA \| 347967 \| \| BV605 \| anti-human CD62L \| DREG-56 \| BD Pharmingen \| Franklin Lakes, NJ, USA \| 562719 \| \| APC \| anti-human CD14 \| MfP9 \| BDIS \| Franklin Lakes, NJ, USA \| 652833 \| \| BV421 \| anti-human CD19 \| HIB19 \| BioLengend \| San Diego, CA, USA \| 302234 \| \| BV510 \| anti-human CD56 \| HCD56 \| BioLengend \| San Diego, CA, USA \| 318340 \| \| APC \| anti-human CD4 \| OKT4 \| BDIS \| Franklin Lakes, NJ, USA \| 663498 \| \| PE/cyanine7 \| anti-human CD8 \| SK1 \| Agilent \| Santa Clara, Calif, USA \| 8931024 \| \| PE \| anti-human CD45RO \| UCHL1 \| BDIS \| Franklin Lakes, NJ, USA \| 347967 \| \| BV605 \| anti-human CD62L \| DREG-56 \| BD Pharmingen \| Franklin Lakes, NJ, USA \| 562719 \| \| PerCP \| anti-human CD8 \| SK1 \| BDIS \| Franklin Lakes, NJ, USA \| 347314 \| \| AF488 \| anti-human EGFR \| AY13 \| BioLengend \| San Diego, CA, USA \| 352908 \| \| APC \| anti-human CD107a \| H4A3 \| BioLengend \| San Diego, CA, USA \| 328620 \| \| Pacific Blue \| anti-human CD3 \| SK7 \| BioLengend \| San Diego, CA, USA \| 344824 \| \| BV785 \| anti-human CCR6 \| G034E3 \| BioLengend \| San Diego, CA, USA \| 353421 \| \| APC \| anti-human CD45RO \| HCHL1 \| BioLengend \| San Diego, CA, USA \| 304210 \| \| BV605 \| anti-human CD62L \| DREG-56 \| BD Pharmingen \| Franklin Lakes, NJ, USA \| 562719 \| \| BB515 \| anti-human CD19 \| HIB19 \| BD Horizon \| Franklin Lakes, NJ, USA \| 564456 \| \| PE-CY7 \| anti-human CD56 \| NCAM16.2 \| BDIS \| Franklin Lakes, NJ, USA \| 663487 \| \| V500 \| anti-human CD45 \| HI30 \| BD Pharmingen \| Franklin Lakes, NJ, USA \| 560777 \| \| PE \| anti-human CD19 \| HIB19 \| BD Pharmingen \| Franklin Lakes, NJ, USA \| 302208 \|  3.2 Supplementary Table 3 supplementary Table 3. Clinical characteristics of patients with different MIP3α expression levels in training and validation cohort | | | | | | | | |
| --- | --- | --- | --- | --- | --- | --- | --- | --- | --- | --- | --- | --- | --- | --- | --- | --- | --- | --- | --- | --- | --- | --- | --- | --- | --- | --- | --- | --- | --- | --- | --- | --- | --- | --- | --- | --- | --- | --- | --- | --- | --- | --- | --- | --- | --- | --- | --- | --- | --- | --- | --- | --- | --- | --- | --- | --- | --- | --- | --- | --- | --- | --- | --- | --- | --- | --- | --- | --- | --- | --- | --- | --- | --- | --- | --- | --- | --- | --- | --- | --- | --- | --- | --- | --- | --- | --- | --- | --- | --- | --- | --- | --- | --- | --- | --- | --- | --- | --- | --- | --- | --- | --- | --- | --- | --- | --- | --- | --- | --- | --- | --- | --- | --- | --- | --- | --- | --- | --- | --- | --- | --- | --- | --- | --- | --- | --- | --- | --- | --- | --- | --- | --- | --- | --- | --- | --- | --- | --- | --- | --- | --- | --- | --- | --- | --- | --- | --- | --- | --- | --- | --- | --- | --- | --- | --- | --- | --- | --- | --- | --- | --- | --- | --- | --- | --- | --- | --- | --- | --- | --- | --- | --- | --- | --- | --- | --- | --- | --- | --- | --- | --- | --- |
|  |  | Training cohort | | | Validation cohort | | | *P* value (training cohort versus validation cohort) |
|  |  | Low level (n = 23) | High level (n = 20) | *P* value | Low level (n = 17) | High level (n = 13) | *P* value |  |
| **Age** | |  |  |  |  |  |  |  |
|  | Years, median (range) | 40 (17-67) | 47 (16-61) | *0.147* | 43 (15-65) | 48 (11-65) | *0.773* | *0.844* |
| **Sex** | |  |  |  |  |  |  |  |
|  | Female | 9 (39.1) | 9 (45.0) | *0.697* | 8 (47.1) | 5 (38.5) | *0.921* | *0.900* |
| **Diagnosis** | |  |  |  |  |  |  |  |
|  | B-ALL | 6 (26.1) | 6 (30.0) | *0.775* | 6 (35.3) | 4 (30.8) | *1.000* | *0.619* |
|  | ph or ph-like + | 0 (0.0) | 1 (16.7) | *1.000* | 3 (50.0) | 2 (50.0) | *1.000* | *0.088* |
|  | ph - | 6 (100.0) | 5 (83.3) |  | 3 (50.0) | 2 (50.0) |  |  |
|  | B cell Lymphoma | 17 (73.9) | 14 (70.0) |  | 11 (64.7) | 9 (69.2) |  |  |
|  | Double hit/Triple hit | 1 (5.9) | 1 (7.1) | *1.000* | 1 (8.3) | 0 (0.0) | *1.000* | *1.000* |
|  | None | 16 (94.1) | 13 (92.9) |  | 11 (91.7) | 9 (100.0) |  |  |
| **Prior HSCT** | |  |  |  |  |  |  |  |
|  | Autologous | 5 (21.7) | 3 (15.0) | *0.523* | 1 (5.9) | 1 (7.7) | *0.922* | *0.151* |
|  | Allogeneic | 1 (4.3) | 0 (0.0) |  | 2 (11.8) | 1 (7.7) |  |  |
| **Prior treatment** | |  |  |  |  |  |  |  |
|  | First line | 1 (4.3) | 2 (10.0) | *0.740* | 2 (11.8) | 4 (30.8) | *0.167* | *0.197* |
|  | Sencond line | 7 (30.4) | 5 (25.0) |  | 4 (23.5) | 5 (38.5) |  |  |
|  | ≥Third line | 15 (65.2) | 13 (65.0) |  | 11 (64.7) | 4 (30.8) |  |  |
| **Refractory or relapse** | |  |  |  |  |  |  |  |
|  | Refractory | 5 (21.7) | 7 (35.0) | *0.624* | 7 (41.2) | 5 (38.5) | *0.789* | *0.202* |
|  | First relapse | 10 (43.5) | 7 (35.0) |  | 4 (23.5) | 2 (15.4) |  |  |
|  | ≥Second relapse | 8 (34.8) | 6 (30.0) |  | 6 (35.3) | 6 (46.2) |  |  |
| **CNS involvement** | |  |  |  |  |  |  |  |
|  | Yes | 0 (0.0) | 1 (5.0) | *0.944* | 1 (5.9) | 4 (30.8) | *0.187* | *0.078* |
| **CAR-T cell dose, ×106 cells/kg, median (range)** | |  |  |  |  |  |  |  |
|  | CART19 | 4.00 (2-10) | 4.00 (2-8.6) | *0.642* | 3 (1-8) | 5.19 (2-8.86) | *0.167* | *0.942* |
|  | CART22 | 5.80 (2-10) | 4.00 (1-10) | *0.142* | 5.55 (1-11) | 5.27 (1-11.35) | *0.981* | *0.790* |
| **Inflammatory factor level** | |  |  |  |  |  |  |  |
|  | IL-6 max, median (range) | 324.8 (46.9-5000) | 269.8 (83.3-5000) | *0.808* | 154.7 (27.1-5000) | 324.8 (46.9-5000) | *0.457* | *0.602* |
|  | Ferritin max, median (range) | 3565.1 (412.6-20349) | 2887.7 (219.2-50000) | *0.158* | 2955.7 (439.4-8526.3) | 3565.1 (412.6-20349) | *0.213* | *0.537* |
| **CRS grading** | |  |  |  |  |  |  |  |
|  | Grade 0-2 | 18 (78.3) | 18 (90.0) | *0.531* | 16 (94.1) | 10 (76.9) | *0.406* | *0.989* |
|  | Grade 3-5 | 5 (21.7) | 2 (10.0) |  | 1 (5.9) | 3 (23.1) |  |  |
| **Sampling time*** | |  |  |  |  |  |  |  |
|  | Days, median (range) | 5 (1-10) | 4.5 (3-7) | *0.347* | 5 (2-10) | 4 (2-8) | *0.805* | *0.799* |
| **Follow-up time** | |  |  |  |  |  |  |  |
|  | Days, median (range) | 142 (9-961) | 854 (61-990) | *0.022* | 100 (14-987) | 410 (63-1005) | *0.010* | *0.059* |
| *****Days after CAR T-cell infusion with the peak of CRS, which was also the day the serum specimen was collected for detection. | | | | | | |  |  |

## 3.3 Supplementary Table 4

| **Supplementary Table 4. *P*-values of cytokines in B-ALL and B-NHL cohorts** | | | |
| --- | --- | --- | --- |
| B-ALL | | B-NHL | |
| Cytokines | *P* value | Cytokines | *P* value |
| **MIP3α** | *0.024* | **MIP3α** | *0.048* |
| **Eotaxin** | *0.042* | **Eotaxin** | *0.019* |
| IL-17A | *0.006* | MCP-4 | *0.003* |
| IL-18 | *0.024* | EPO | *0.025* |
| ENA-78 | *0.042* | TSLP | *0.033* |
| IL-5 | *0.042* | Eotaxin-3 | *0.037* |
| G-CSF | *0.042* | MDC | *0.037* |
| IL-12 | *0.042* | IL-10 | *0.042* |
| TPO | *0.042* |  |  |

# 4 Supplementary References

1. Wang N, Hu XL, Cao WY, Li CR, Xiao Y, Cao Y, et al. Efficacy and safety of CAR19/22 T-cell cocktail therapy in patients with refractory/relapsed B-cell malignancies. Blood. 2020;135(1):17-27.

2. Cheson BD, Fisher RI, Barrington SF, Cavalli F, Schwartz LH, Zucca E, et al. Recommendations for Initial Evaluation, Staging, and Response Assessment of Hodgkin and Non-Hodgkin Lymphoma: The Lugano Classification. Journal of Clinical Oncology. 2014;32(27):3059-67.

3. Lee DW, Gardner R, Porter DL, Louis CU, Ahmed N, Jensen M, et al. Current concepts in the diagnosis and management of cytokine release syndrome. Blood. 2014;124(2):188-95.

4. Brudno JN, Kochenderfer JN. Toxicities of chimeric antigen receptor T cells: recognition and management. Blood. 2016;127(26):3321-30.

5. Mandrekar JN. Receiver Operating Characteristic Curve in Diagnostic Test Assessment. J Thorac Oncol. 2010;5(9):1315-6.

6. Youden W. Index for rating diagnostic tests. Cancer. 1950;3(1):32-4.
